# Design of an orthopaedic-specific discharge summary

**DOI:** 10.1186/s12913-016-1783-x

**Published:** 2016-10-04

**Authors:** Christine Soong, Bochra Kurabi, Kathleen Exconde, Faiqa Tajammal, Chaim M. Bell

**Affiliations:** 1 Division of General Internal Medicine, Mount Sinai Hospital, 600 University Avenue, Room 428, Toronto, ON M5G 1X5 Canada; 2 Institute of Health Policy, Management and Evaluation, Toronto, ON Canada; 3 Department of Medicine, University of Toronto, Toronto, ON Canada; 4 Inter-department Division of Critical Care Medicine, University Health Network, Toronto, ON Canada

**Keywords:** Hip fracture, Comanagement, Hospitalist, Quality improvement

## Abstract

**Background:**

Patients undergoing orthopaedic procedures experience major changes in function and daily routines upon their return home. Discharge summaries are an important communication tool that may play a role in optimizing a safe transition from hospital. Current care gaps and key elements of an ideal discharge summary specific for orthopaedic population are unknown. We sought to identify the challenges of current orthopaedic discharge summaries and to determine key elements of an ideal document.

**Methods:**

Qualitative study survey using semi-structured interviews with a sample of 17 patients and clinicians representing diverse professions, backgrounds, and practice settings. We used the constant comparative method of qualitative analysis to define the experiences and perceptions of quality gaps and strategies to improve orthopaedic-specific discharge summaries.

**Results:**

We identified 3 major themes describing factors perceived to be limiting the quality of current discharge summaries: 1) physician-centric documentation and the absence of a comprehensive, inter-professional perspective; 2) access to resources and health informatics; and 3) process variations in document creation and dissemination.

**Conclusions:**

Clinicians and patients identified several factors limiting the quality of discharge summaries among orthopaedic inpatients. Incorporating these elements could improve hospital transitions.

## Background

Hospitals are facing intense pressure to increase efficiency and reduce length of stay in the face of rising patient complexity. Not surprisingly, there is an increase in the volume of patients discharged to post-acute care as a solution for those who no longer require high intensity care but are unprepared for home [1]. Patients returning home or discharged to post-acute care facilities are equally exposed to preventable harm, often as a result of suboptimal transitions of care. While poor transitions have been extensively studied for patients returning home, little is known of the quality of inter-facility transitions and the new problems that may arise as a result. What *is* known, is that patients experiencing multiple transitions across the continuum of care are at risk of preventable harm due to suboptimal communication between providers [2]. In particular, gaps in information transfer may contribute to adverse events such as delayed actionable test results and readmission rates [3, 4].

High quality discharge summaries play an important role in transferring critical information between providers and may reduce adverse events (such as avoidable readmission) in the post-discharge period [5]. Studies examining the quality of discharge summaries for patients discharged to sub-acute facilities suggest there is room for improvement [6, 7]. In particular, details on activity instructions, therapy orders, and pending studies are frequently missing from discharge summaries created for patients transferred to rehabilitation facilities [6].

Patients undergoing orthopaedic procedures such as joint replacement and hip fracture surgery frequently experience significant changes to their function and performance status. High quality discharge summaries can play an essential role in reducing post-discharge adverse events and ensuring appropriate transfer of information to outpatient and post-acute care facility providers. However, gaps specific to current orthopaedic discharge summaries have yet to be characterized and best practices remain unclear.

We conducted a qualitative study of key stakeholders to identify specific barriers to creating a high quality orthopaedic discharge summary and to determine key elements of an ideal document. This study was conducted at Mount Sinai Hospital in Toronto, Canada, a 442-bed acute care academic health sciences center affiliated with the University of Toronto. In 2014–15, there were 883 patients admitted to the orthopaedic service consisting of both elective and emergency procedures. Within the division of orthopaedic surgery, there are 7 faculty members. Currently, discharge summary creation differs among patients on the orthopaedic service. For patients undergoing elective joint replacement, 1 of 7 residents rotating on the service dictates an unstructured discharge summary. Patients with hip fractures are co-managed by hospitalists who complete an electronic discharge summary template in lieu of a dictated summary.

## Methods

### Study design and participants

This qualitative study used semi-structured interviews of 17 patients and clinicians representing diverse professions, backgrounds, and practice settings (Table 2).

Participants were selected based on their involvement in the care of orthopaedic patients along the acute care to post-acute care continuum. Specifically, participants were eligible if they 1) provided care to patients undergoing orthopaedic procedures in hospital (acute care, or post-acute care facilities), or 2) if they or their loved one was hospitalized to undergo an orthopaedic procedure. We used a purposeful and snowballing sampling approach to recruiting participants [8]. We began with nursing leaders from both the acute care and rehabilitation facilities to identify potential interviewees. This approach allowed for diverse and representative perspectives. Each participant provided informed consent. The Mount Sinai Hospital Research Ethics Board approved this study.

### Interview process

Interviews were conducted in an office setting within a hospital. We used an open-ended interview guide to elicit clinicians’ experiences around challenges to effective inter-facility care transitions and to identify ways to optimize practice. Following a literature review of discharge summary key issues and suggested best practices, we created the interview guide and prompts to capture orthopaedic-specific challenges and relevance within our context (Table 1). The interview guide was pilot tested among 3 clinicians to ensure clarity. A research assistant trained in qualitative methods conducted the interviews which were then transcribed.

**Table 1:** Interview guide

Interview question	Prompt
What is your experience with the current orthopaedic discharge summary?	Have you experienced any deficiencies or challenges while reading or writing a discharge summary? (If yes, please describe)
	What do you view as redundant or minimally useful components of the current discharge summary?
What would you like to see changed with a new electronic discharge summary template?	What do you see as valuable elements of a high quality orthopaedic-specific discharge summary? (ie. Main diagnosis, pertinent physical findings, results of procedures, pain management, DVT prophylaxis, wound care, follow up care? Appendix 1 shown to participants)
In your opinion, what logistical and environmental barriers might affect the completion of an ideal electronic discharge summary?	What methods or procedures could be amended to implement the revised orthopedic discharge summary?

To understand which elements of a discharge summary would be felt to be valuable, we used a template created in collaboration with our regional peer hospitals that outlined suggested elements to be included in discharge summaries. This template was shown to participants at the end of the interview (Table 4 in Appendix).

### Data analysis

A research coordinator anonymized and transcribed the interviews prior to analysis. We used a constant comparative approach, consistent with grounded theory methodology [8]. One investigator (B.K.) read the initial transcripts during the open coding process and identified emerging themes and developed the coding scheme. Analysis focused on identifying the challenges of current orthopaedic discharge summaries that were perceived by participants and to identify key elements of an ideal document. The transcripts and coding scheme were then reviewed by another investigator (C.S.) to ensure reliability. The process of open coding for emergent themes proceeded iteratively, with the coding structure constantly being refined to identify conceptual segments of data based on additional interview transcripts. Once theoretical saturation was reached, the coding scheme was deemed stable. Data and codes were organized using N-vivo® software Version 8 (Cambridge, MA, US), a qualitative data management software package.

## Results

Out of 18 e-mail invitations, we successfully recruited 17 participants. Our findings revealed a number of common themes among participants, all of whom have had a range of experience (comprehensive, minimal, and zero experience) with the current orthopaedic discharge summary in their various roles in the hospital and rehabilitation setting (Table 2).

**Table 2:** Participant (patient and provider) characteristics

Patients	*N* = 2 (%)
Mean age, y (SD)	68.5 (23)
Male	2 (100)
Mean # of times admitted	1.5
Reason for admission	
Traumatic fracture	1 (50)
Elective arthroplasty	1 (50)
Surgery performed	2 (100)
Providers	N = 15 (%)
Mean age, y (SD)	40.5 (6)
Male	7 (47)
Profession	
Pharmacist	1 (6.7)
Hospitalist	1 (6.7)
Physiotherapist	1 (6.7)
Geriatrician	2 (13.4)
Social worker	1 (6.7)
Nursing unit administrator	1 (6.7)
Orthopaedic surgeon	1 (6.7)
Occupational therapist	1 (6.7)
Family physician	3 (20)
Geriatric psychiatrist	1 (6.7)
Nurse	1 (6.7)
Health services researcher	1 (6.7)
Work setting	
Hospital	12 (80)
Primary care office affiliated with a hospital	3 (20)

### Challenge of current of discharge summary

Three major themes emerged from the interviews describing current challenges to producing high quality discharge summaries for orthopaedic inpatients. These include 1) physician-centric documentation; 2) access to resources and health informatics technology; and 3) process variations.

1. Physician-centric documentation

Participants identified the lack of accurate, representative, and consistent information about the patient’s hospital stay as a major challenge. Currently, hospital processes place the burden on one health care provider (the physician) to complete the orthopaedic discharge summary, yet this individual may lack a comprehensive perspective to all aspects of a patient’s care plan. Typically, there are multiple clinicians involved in the care of a single patient. However, their limited access to discharge summaries decreases the quality of the information provided. The result is an incomplete picture of the patient’s hospital stay and health management plan. The following narratives illustrate this theme.

“Maybe some nursing input, allied health input, along with the medical staff’s input [is needed]. …it’s not just medical issues, there are lots of social issues involved as well, especially in the hip fracture population. So if [sic] their prehospital status– were they…independent with activities of daily living, where did they come from, did they come from home, did they come from a facility, what kind of facility, their functioning status and stuff like that − so to have a…broader picture and not just a surgical picture of a specific injury and a specific treatment for that injury.” – **Nursing administrator**

“Well the barriers are…what I mentioned. You might have to write about things that you don’t know about. For example, if the surgeon is writing about delirium, he or she is not going to get it right. And if I have to write about the actual hip fracture or surgery, I’m not going to get it right and I may not know as much as they would. So one barrier is expertise – we are filling out discharge summaries for things that we were not directly involved in. That is why discharge summaries are everyone’s job or duty.” – **Geriatrician**

“I know you can’t write a book about everything but I think sometimes we leave out important things on discharge summaries. Every person views important things differently, which is why everyone should be involved in writing a discharge summary.”– **Geriatrician**

“So they probably need to liaise with [the] pharmacy medication management system to incorporate that and make sure the medications are structured and formatted in a clear way.”– **Geriatrician**

“Maybe [with] multiple people involved in the care of the patient, they might not necessarily know all the details of the patient, so might leave things out. So again, if a patient’s admission straddled the change of a rotation, maybe the resident or the doc that admitted the patient or did the surgery isn’t the same person that’s discharging them. So maybe they won’t know all of the fine details of the history and the details of the stay.” – **Family physician**

“Consistency, number one…that the orthopaedic surgeons can use it, the hospitalists can use it, other services can use it, maybe even gen surg, so that everyone is talking in the same language. Everyone speaks to a discharge summary; they know what it is, they know what to expect.”– **Clinical nurse specialist**

“For example, we are not routinely consulted as geriatricians or geriatric psychiatrists in the development of that discharge summary. We certainly do dialogue with our hospitalist colleague about issues of concerns, and I do think some of the issues…will be reflected in the discharge summary the hospitalist will be doing, but I think we are not in the formal part of the discharge process. So, for example, there may be an issue that we feel is important or worthy of mentioning that the hospitalist may not have thought was worthy of mentioning…For example, bone health management or various disposition planning issues. I think they are…more likely to be mentioned now than they were ever before and I think timely information exchange is occurring, but…working in the rehab setting, I certainly do see inadequate discharge summaries…In general, there is still kind of a desire that’s needed for the receiving facility to really optimize the care being provided in the next phase.” – **Geriatrician**

Participants felt handwritten discharge summaries posed a barrier in legibility and frequently contained inadequate information. Redundant information was viewed as a challenge to some who felt information was already provided in the electronic medical record (EMR). On the contrary, others felt that although redundant information was bothersome, more information was better “in case” the end-user might find it relevant.

“Usually people can read through the important and unimportant things” – **Geriatrician**

“I don’t think there is anything I would say is redundant or minimal use [sic]. Particularly if I am an [end]-user and receiving the discharge summary, I generally look at all of the sections so to me they’ve all been fairly useful. I can’t think and say consistently one area is redundant or not useful.” – **Geriatrician**

2) Access to resources and health informatics

Another challenge described by participants was inadequate resources to complete discharge summaries in a timely fashion. This resulted in delayed transfer of information to physicians within a patient’s circle of care. Furthermore, participants described settings with a hybrid of paper and electronic medical records. In this scenario, information is gathered from multiple sources resulting in barriers to the timely completion of the discharge summary.

“So it’s often hard to find a quiet place to actually sit down and do the work. So that is one barrier sometimes. And it’s mostly time. Time is the hard one.”– **Clinical nurse specialist**

“I found that the discharge summary does not seem to accompany the patient to their next destination. For example, sometimes I’ll see the same patients when they go to…rehab as an inpatient because I sometimes spend time there. Occasionally I’ll see the patients when they are seen in my clinic in follow up and I don’t always get a copy of the discharge summary, so sometimes I have to go and find it on my own or else the discharge summary may come late. So it doesn’t always accompany the patient. I have that as one challenge.”– **Geriatrician**

Health information technology (IT) challenges were cited as another barrier to the creation of a high quality discharge summary. Participants described slow computers, and the inability to edit or save a document that was simultaneously being edited by another health care provider. In addition, currently discharge summaries are faxed rather than sent electronically to primary care physicians (PCP). The ability to prepopulate existing information such as results of bloodwork, PCP information, previous medication records, and prescriptions would contribute to the timely completion of the discharge summary. Another identified IT solution was electronic prompts to the provider to complete the discharge summary.

“You need an adequate IT department to again, draw the data into the summary, rather than having to dictate it and re-digest it every single time.” –**Rehabilitation hospitalist**

“I think the technology that is in place probably needs to be reviewed and rationalized to a point to make accessing certain pages within Power Chart a little easier and getting rid of duplication that is involved in some of the processes. Just making it easier to use essentially, I think that’s it.” – **Nurse administrator**

“…If you are on a patient’s record…you can fill in their discharge summary on just EPR [Electronic Physician Record] and not going to a separate system to do it. And also it could even flag you every time you log in. For example, “Mr. X is leaving in 2 days, have you thought about doing a discharge summary?” Or it should even prompt you to do it right away like “Mr. X is leaving tomorrow, you have not done the discharge summary. Start working on it.” So that might be a nice little reminder. Because when you are there, you are thinking about the patient. …[If] not, you know— you are then rushing to do it because the patient is leaving in ten minutes. When you are on the system, e.g., “oh, you are looking at his labs anyway so…” Your memory is jogged, you are thinking about it, just put it in to the system.” – **Geriatrician**

3) Process variation

Different processes of discharge summary creation and dissemination were seen as a challenge by the participants. Many participants were uncertain who received copies of the discharge summary. Some suggested forming a relationship with post-acute care facilities to better understand whether information is received in a timely manner and what aspects of the process can be improved.

“The biggest problem I have is that there is no standardization with the process amongst all the hospitals and we sort of have to know what’s going on based on which hospital they come from, which surgeon they’re seeing, and I mean after time you get to know one from the other and everybody’s nuances.” – **Rehabilitation hospitalist**

“I think, number two— I don’t think it’s the writing of the discharge summary that’s the difficulty, I think it’s more of, if we say yes, these elements are important, then we need to create a bit of support of a process there— that “hey Mrs. Smith has a bed, she should be going in 2 days, anything you want to add in?” and create a process for people who can go in and contribute into that discharge summary. So we need to create a workflow around that. So at least if we have 24 h of advance notice we can make sure we put that in place, otherwise we have to write separate letters and send them off afterwards. But if we know that the patient is going then we can at least make sure that those key elements are added in, so that it doesn’t necessarily fall on one person to have to always do.” – **Geriatrician**

### Valuable elements

Table 3 displays the identified valuable elements of an orthopaedic-specific discharge summary. Participants commented on the need for detailed timelines and format of the discharge summary. Areas of high priority unique to orthopaedic patients include falls, bone health, functional status and cognitive status. In general, participants identified the templated discharge summary as a valuable tool to characterize the patient’s hospitalization. The participants consistently identified the same list of “valuable elements” for an orthopaedic-specific discharge summary. 100 % of participants agreed with the elements included in Table 3, with the exception of falls history that was suggested by 3/17 participants.

**Table 3:** Valuable elements of a discharge summary

Admitting diagnosis
Final diagnosis
Admission and discharge date
Contact information
- Primary care provider (name, phone, fax) - Hospital physician or resident - Specialists
Detailed patient history
- Medical/surgical - Daily living (social, mobility) - Home environment
Course in hospital
- Surgery: Concise summary including date of surgery, type of surgery and selection of anaesthesia; Preoperative care; Postoperative care and complications - Adverse events: procedures required (detailed); delirium, Date occurred; Management, Pertinent negatives - Other conditions impacting hospital stay (medical/surgical/social) - Goals of care: code status, advanced directives, - Functional status: behaviour, precautions or restrictions and participation at discharge (Include PT and OT documentation) - Cognitive function and assessments pre and post discharge
Falls
- History of falls: frequency, mechanism - Details about the fall that precipitated fracture - Falls prevention recommendations
Bone health
- Bone health investigations - Bone health management - Changes to osteoporosis medications – initiation, dosing and follow-up implications.
Consult services and recommendations
- Rehabilitation therapists (details on consent, assessment and treatment) - Geriatric medicine, geriatric psychiatry, medical consults, anesthesia, etc
Summary of key results, investigations, summary of bloodwork to identify trends
Medication
- Allergies - Medications reconciliation - Pain control - Venous thromboembolism prophylaxis - Osteoporosis treatment - Adherence: # of tablets
Post-discharge health management plan
- Follow-up appointments (those made or to be made, including dates and times) - Health management plan and timelines – clear instructions to patient and primary care provider - Outstanding Investigations and pending results - Referrals and homecare supports - Projections and expectations on recovery - Discharge facility (unit and program) - Wound care instructions - Equipment required pre and post hospital discharge

## Discussion

We identified 3 major themes describing factors perceived to be limiting the quality of current discharge summaries for patients undergoing orthopaedic procedures. These include: physician-centric documentation and the absence of a comprehensive, inter-professional perspective; access to resources and health informatics; and process variations in document creation and dissemination. Addressing these barriers may improve the quality of discharge summaries.

The first theme of discharge summaries missing accurate information reflecting all perspectives of inter-professional team members is a recurring one. Several studies of discharge summaries have demonstrated poor information transfer [9, 10]. Horwitz and colleagues reported physicians were failing to capture relevant details from other members of the inter-professional team [11]. Jeffs and colleagues described hospital and post-acute care clinicians’ views on safe transfers and uncovered a similar theme [12]. Leveraging EHR to pull information documented by multiple providers for the same patient could generate a more comprehensive summary. Our study participants suggested a true inter-professional approach to documentation by inviting other clinical team members to contribute to discharge summary creation. This has important implications for the current practice where it is common to have a single provider to complete a document reflecting recommendations provided by multiple health care providers.

Our second theme of access to resources and health informatics has been described as a common barrier to quality discharge summaries [13]. EHR can enhance discharge summaries in several ways such as improved efficiency, comprehensiveness of documents, and greater PCP satisfaction [13–15]. Furthermore, electronic discharge summary creation has been associated with improved user satisfaction without significantly increasing time burden [14]. Electronic discharge summary software programs may be used to address current gaps in EHR systems unable to accommodate discharge summary templates [14].

Our third theme of process variation and lack of standardization of discharge summary creation and dissemination is associated with poor information transfer and low PCP satisfaction [9]. Experts call for standard components to be included in each discharge summary [16–18]. EHR and templated discharge summaries can enable structured information transfer and increase standardization. Providers should receive appropriate training on discharge summary documentation and hospitals should provide the necessary infrastructure and resources to achieve these goals.

This is among the initial work to provide clarity on key aspects essential to an orthopaedic-specific discharge summary. Other studies on discharge summaries have focused on medical inpatients with different challenges and needs [16, 19, 20]. In contrast to other studies, our sample included patients, reflecting comprehensive and diverse viewpoints. Our participants identified important elements frequently missing on generic discharge summary templates that should be included for patients undergoing orthopaedic procedures. Design of a novel discharge summary would improve the transfer of critical information to PCPs and post-acute care rehabilitation providers. This is the first step in the creation of a high-quality discharge document.

Several limitations of this study merit discussion. First, as a single-centre and qualitative study, our results are subject to limited generalizability. Second, our findings are limited to the views of selected participants and may lack comprehensive perspectives. Still, our sampling method was chosen to be inclusive in order to capture all possible viewpoints, including those of patients. Moreover, we utilized a standard qualitative approach whereby we continue with interviews until we reach theme saturation.

## Conclusions

Our study revealed a highly variable documentation process lacking in complete inter-professional information capture in discharge summary creation for orthopaedic patients. There is an opportunity to standardize the transfer of information for a vulnerable population undergoing significant functional changes. Future efforts should focus on interventions that increase adherence to discharge summary template use and EHR-enabled strategies to capture inter-professional provider assessments and recommendations. Finally, the implementation of a tailored discharge summary addressing common issues experienced by orthopaedic patients should be explored. A patient-centred, inter-professional discharge summary is the first step towards ensuring safe transitions and improved quality of care for orthopaedic patients.

## Appendix

**Table 4:** Discharge summary template elements

Patient demographics	Patient name, MRN
	Date of birth
	Gender
	Primary care provider
Visit	Admit date
	Discharge date
	Most responsible health care provider name and contact information
	Name of individual completing summary
	Date completed
	Discharge location
	Death (yes, no)
Encounter location	Hospital name
	Hospital type
Alert indicators	Allergies
Course while in hospital	Presenting complaint(s)
	Summary of key results, investigators, interventions, and advance directives
	Adverse events and complications
Discharge plan	All medications at discharge
	Follow-up instructions for patient
	Follow-up plan recommended to be implemented by the receiving provider
	Referrals
	Copied to with contact information

## Abbreviations

EHR: Electronic health record
PCP: Primary care provider
